# The sex gap among visitors during flexible intensive care unit visiting hours

**DOI:** 10.5935/0103-507X.20190089

**Published:** 2019

**Authors:** Silvana Pinto Hartmann, Larissa Jorge F. de Faria, Cassiano Teixeira, Cristiane Souza dos Santos, Tiago Claro Maurer, Daiana Barbosa da Silva, Regis Goulart Rosa

**Affiliations:** 1 Unidade de Terapia Intensiva, Hospital Moinhos de Vento - Porto Alegre (RS), Brasil.; 2 Programa de Pós-Graduação em Ciências da Reabilitação, Universidade Federal de Ciências da Saúde de Porto Alegre - Porto Alegre (RS), Brasil.; 3 Faculdade de Medicina, Universidade Federal de Ciências da Saúde de Porto Alegre - Porto Alegre (RS), Brasil.

**Dear Editor,**

Flexible intensive care unit (ICU) visiting hours are increasingly recognized as a means of enhancing patient- and family-centered care.^(1,2)^ In addition to being safe and associated with better patient and family outcomes,^(3,4)^ flexible visiting hours are an important approach to acknowledging and showing respect for the patient-family relationship during the course of critical illness.^(1,2)^ In this context, knowledge of visitor characteristics is essential to determining the best way to support them and to promote the sustainability of flexible visitation models (FVMs). Particularly, the assessment of sex differences among visitors is necessary, since gender-specific factors may influence the demand for tailored communication strategies, the sharing of the decision-making process, and the prevention of psychological burdens. Therefore, we conducted this study to investigate the existence of a sex gap among visitors of critically ill adult patients during an FVM.

**Figure 1:**
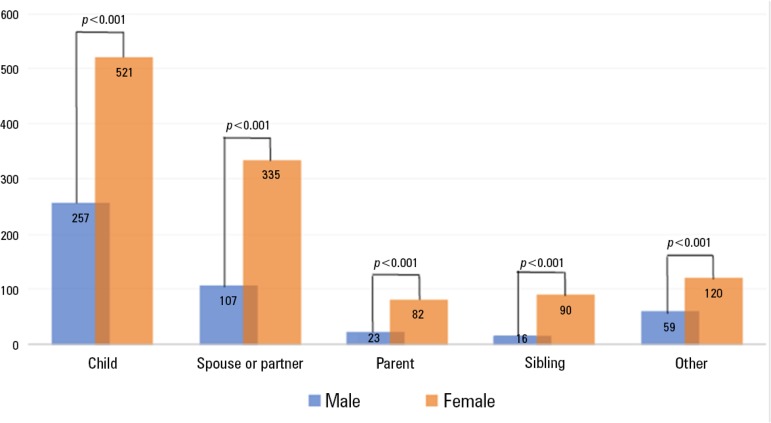
Distribution of intensive care unit family visitors according to sex and kinship category. The data shown are the number of visitors. The "other" category includes family members from other kinship categories (e.g., grandparent, grandchild, aunt/uncle, cousin, stepparent, stepchild). The chi-square goodness-of-fit test was used to determine whether the frequency distribution of sex in each kinship category followed the hypothesized male-female ratio of 1:1.

A cross-sectional study was performed in a single 48-bed medical-surgical adult ICU of a tertiary hospital in southern Brazil from January to October 2018. During this period, close family members were allowed to visit the critically ill patient for up to 12 hours/day. To join the FVM, visitors had to attend an educational meeting designed to help them understand the structural and organizational aspects of critical care (Table 1). All consecutive family members who agreed to join the FVM and who participated in the educational meeting during the study period were included in this analysis. The study was approved by the institutional review board of *Hospital Moinhos de Vento* . Of the 1610 family members assessed, 1148 (71%) were women. In all kinship categories, women visited more frequently than men (Figure 1).

**Table 1:** Description of the intensive care unit visitor educational meeting

	Description
Objective	Education of family visitors with the aim to improve their understanding of the structural and organizational aspects of critical care
Target population	Family visitors of ICU patients
Frequency	Once a day, seven days per week
Format	Face-to-face meeting conducted by trained health care professionals
Topics addressed	ICU environment, common ICU treatments, rehabilitation and basic infection control practices, multidisciplinary work at the ICU, palliative care, and delirium prevention

ICU - intensive care unit.

The higher proportion of females among ICU visitors may be explained by a set of social, cultural, and psychological factors. First, women are traditionally [often] expected to care for sick family members. Accordingly, the specific social and cultural circumstances experienced by women or men from childhood to adulthood may lead to distinct reactions to the caregiver role. Second, the existing sex inequality in the labor market may contribute to the social pressure on women to undertake the role of ICU visitor; since women are less likely to have a job outside the home, they would be available for ICU visits. Third, there are sex differences in coping with stress, which may influence how women and men face the critical illness of a loved one. While women are more likely to have emotion-focused coping styles (and more emotional stress as well), men more often have rational and detached coping styles.^(5)^ These differences may ultimately contribute to the predominance of women among ICU visitors.

In conclusion, our results showed a sex gap among visitors during flexible ICU visiting hours, with women predominantly undertaking the role of family visitors. This finding draws attention to the importance of improving female-focused support strategies for visitors in ICUs with flexible visiting hours.
